# Editorial

**Published:** 1899-03

**Authors:** 


### EDITORIAL.

### THE ARMY BILL.

We print elsewhere a record of the legislation in regard to army veterinarians which has been enacted by the Fifty-fifth Congress. The provisions worded by the subcommittee of the Senate have become a law for the present. The "pay and allowances of a second lieutenant of cavalry" have been granted to the senior veterinarian of each cavalry regiment, and the second veterinarian is granted seventy-five dollars a month and the "allowances of a sergeant-major."

With this a strong point has been gained in the requirement of an examination for appointment, which does away with the absolutely unrecognized character of the veterinary surgeon's position which we have had heretofore, and insures a reputable professional rating for those who enter in the future.

We have the satisfaction, at last, of seeing the work of the Committee on Army Legislation of the American Veterinary Medical Association meet with some reward, after a dozen years of labor, and feel that the veterinarian is now recognized as a component part of the army.

But this is only an entering wedge, and immediate steps must be taken to secure better legislation in the next Congress.

In each cavalry regiment one veterinarian is assimilated to a commissioned officer, while the second veterinarian is relegated to association with enlisted men. Under these conditions unmarried veterinarians can take the examination for the senior position, with the expectation that a future and better arrangement will be made whereby their previous service will count in advancement. Those who may be willing to take the position of the second veterinarian for seventy-five dollars a month and the "allowances of a sergeant-major" will have to be credited with very great enthusiasm for army life or some very urgent motive.

Look at the position which this arrangement provides for. Veterinarians who have studied for three or more years at a college, and who are peers, as professional men, of the graduate of human medicine or of law, are asked to enter a service where one is assimilated to an officer and the other to an enlisted man. Colleagues in a profession, their social surroundings are absolutely different. This becomes more marked if they should happen to be married. In this case, while the individual man, according to his ability, may make friends with anyone, and the wife of the assimilated second lieutenant may be received by the wives of the officers of a post or garrison, the wife of the junior veterinarian could only associate with the washerwomen and servants of the station. The relation of the veterinarian to the officials of the army is scarcely better to-day than it was. He is simply a contract dependency of the troop, squadron, or cavalry regiment to which he is attached. His only authority will be in the favor granted him by the whim or personal favor of the commander under whom he serves. No provision is made for his equipment and supplies. No means is provided, or asked for, for communication to the headquarters of the army. He remains a contract employé of the government, responsible only to the cavalry commander to whom he is assigned. No provision is made for the good of the general service. The quartermaster-general's work and that of the artillery will still be done by such civilians as they may choose to employ. The record of the service for the last twenty-five years of peace and of the few months of the Spanish-American war has shown us the class of men employed. We have seen thousands of horses, unfit for service, purchased. We have seen glanders in epizoötic form ravaging the corrals of the army until the courts of the State of Florida issued an injunction forbidding the sale of army horses in its precincts, and the State veterinarian of Virginia and the Board of Health of the City of Washington protested against the unloading of diseased animals by the army upon the community.

Every veterinarian in the country should at once appeal to his representatives in Congress, Senators and members and, showing them the present status, secure their influence to help build up a future service which is lacking only in the army of one civilized country in the world—that of the United States of America.

An army veterinary corps and post veterinarian was the tocsin sounded by one of the profession in view of the close of the meeting of Congress.

Senator Hawley, of Connecticut, assured the veterinarians of that State that he was in hearty accord with the proposed measure according to the veterinarian rank in the army service.

The response of the veterinarian to the call for means to defray the expenses of the Army Legislative Committee was prompt and generous.

The Pennsylvania Veterinary Association appropriated sixty dollars from its treasury to the needs of the Army Legislative Committee, while the veterinarians of the State added nearly one hundred dollars more to the cause. The Keystone State veterinarians set a good example to the members of the profession in other States.

Now for the legislation, with recognition and rank that should have been accorded the profession ten years ago, was one way of saying it, as expressed by an army veterinarian.

---

### MARRIAGE.

At Towanda, Pa., February 15, 1899, Dr. Robert G. Rice to Miss Laura A. L'Amoureux. Dr. Rice is a graduate of the New York College of Veterinary Surgeons, class of 1896. He has built up a remunerative practice in Towanda in the past two years.

---

### NECROLOGY.

The death of Mr. M. W. Dunham, of Wayne, Illinois, to whose breeding establishment the members of the United States Veterinary Medical Association were welcomed in 1893, will be sincerely regretted by every one who had the pleasure of meeting him on that occasion. The privilege of looking over the wonderful array of the choicest Percheron and French coach-stallions was one seldom to be enjoyed, and the strong, rugged character, the kindly disposition, and just pride of him whose matchless energy and courage made Oaklawn Farm known the world over will be gratefully remembered by every veterinarian who felt the warm-hearted touch of his welcoming hand that bade us be at home while treading the soil of Oaklawn. Called away at the age of fifty-seven years, in the prime of life, it would seem that he was taken from his life of usefulness and worth too soon; but his good name and remarkable career will stand as an enduring monument ages to come—a tribute to courage, indomitable energy, sound judgment, and success beyond the hope of but few in life.

At Fairport, New York, on February 6, 1899, Dr. Edward D. Burns, graduate of the Ontario Veterinary College, class of 1895, passed away. Dr. Burns' death followed an attack of appendicitis, for which surgical interference proved unavailing. He was a member of the Genesee Valley Veterinary Medical Association.

